# Modified LIX^®^84I-Based Polymer Inclusion Membranes for Facilitating the Transport Flux of Cu(II) and Variations of Their Physical–Chemical Characteristics

**DOI:** 10.3390/membranes13060550

**Published:** 2023-05-24

**Authors:** Fang Hu, Yifa Huang, Yanting Huang, Junming Tang, Jiugang Hu

**Affiliations:** 1 School of Resource Environment and Safety Engineering, University of South China, Hengyang 421000, China; 2 College of Chemistry and Chemical Engineering, Central South University, Changsha 410083, China

**Keywords:** polymer inclusion membrane, hydrophilic modification, membrane permeability, membrane accumulation, impedance spectroscopy

## Abstract

A unique facilitation on the transport flux of Cu(II) was investigated by using modified polymer inclusion membranes (PIMs). LIX^®^84I-based polymer inclusion membranes (LIX^®^-based PIMs) using poly(vinyl chloride) (PVC) as support, 2-nitrophenyl octyl ether (NPOE) as plasticizer and Lix84I as carrier were modified by reagents with different polar groups. The modified LIX^®^-based PIMs showed an increasing transport flux of Cu(II) with the help of ethanol or Versatic acid 10 modifiers. The metal fluxes with the modified LIX^®^-based PIMs were observed varying with the amount of modifiers, and the transmission time was cut by half for the modified LIX^®^-based PIM cast with Versatic acid 10. The physical–chemical characteristics of the prepared blank PIMs with different Versatic acid 10 were further characterized by using attenuated total reflectance Fourier transform infrared spectroscopy (ATR-FTIR), contract angle measurements and electro-chemical impedance spectroscopy (EIS). The characterization results indicated that the modified LIX^®^-based PIMs cast with Versatic acid 10 appeared to be more hydrophilic with increasing membrane dielectric constant and electrical conductivity that allowed better accessibility of Cu(II) across PIMs. Hence, it was deduced that hydrophilic modification might be a potential method to improve the transport flux of the PIM system.

## 1. Introduction

Waste streams containing copper from plating/electrowinning industries are considered to be hazardous materials, which is a great threat to environment [1,2,3]. Liquid membranes (LMs) seem a good alternative to treat these hazardous waste streams, where the LMs are similar to the conventional liquid–liquid extraction. With LMs, extraction and stripping can be performed continuously without the use of large amounts of organic diluents. Various liquid membrane configurations including bulk LMs, emulsion LMs, and supported LMs have been widely explored. For instance, the supported LMs with organic solvent immobilized in micropores of the polymer support have relatively high mass transfer rates, but usually suffer an insufficient membrane stability [4].

As a particular type of LMs, polymer inclusion membranes (PIMs) exhibit excellent stability and versatility owing to the encapsulation of the extractant within the polymer membrane matrix [5,6]. However, PIMs often have low permeability due to the increase in the viscosity of the membrane matrix [7,8]. The diffusion coefficients of Cu species in the PIMs seem to be lower in comparison with supported LMs, which employ hydroxyoximes carriers for copper transport [9,10,11]. PIMs are susceptible to accumulate metal ions within the bulk membrane, thus decreasing the ion permeability performance. Miguel et al. and co-workers [12] reported that the stripping permeability of Cu(II) was obviously lower than the feeding permeability in PIMs because of accumulation in the membranous phase. D. Wang et al. found that the extraction efficiency of Cu(II) was about 80% for LIX^®^84I-based PIM, but its stripping efficiency reduced to only 60% because of the accumulation [13]. The low membranous permeability of PIMs has been reported due to the increasing viscosity of the membranous support medium [7,8]. Recent studies indicated that the membranous components and physico-chemical properties played an important role in the membranous homogeneity, flexibility and ion transport efficiency [14,15]. Generally, PIMs were composed of a base polymer, i.e., poly(vinyl chloride) (PVC) or cellulose triacetate (CTA), an extractant (carrier), and a plasticizer or chemical modifier. Plasticizers and modifiers made a valuable contribution in the transport efficiency of polymer inclusion membranes [16]. Plasticizers, well known in the plastics industry, were used to make PIMs more flexible and to lower the membranous diffusive resistance. The plasticizer molecules could penetrate between polymer strands to increase the distance between the polymer strands so as to reduce the strength of the inter-molecular forces to form homogenous, flexible and mechanically strong membranes [17,18,19]. Chemical modifiers, which were either the extractants or other hydrophobic ligands, were considered as stabilization agents to keep the extracted solute and the extractant soluble in the solid membrane matrix. Although some modifier mixtures have been suggested to facilitate membranous permeabilities, a comprehensive understanding for the modification on the membranous performance is still obscure. It is necessary to figure out the mechanism of membranous modification from physical–chemical characteristic aspects.

With this aim in mind, three modifiers with different polar groups, viz., di(2-ethylhexyl phosphoric acid (D_2_EHPA), ethanol and Versatic acid 10 (V10) were cast into the PIMs to explore the Cu(II) transport flux of PIMs which were prepared by using poly(vinyl chloride) (PVC) as support, 2-nitrophenyl octyl ether (NPOE) as plasticizer, and LIX^®^84I as carrier. The influence of modifiers on metal ion permeabilities of the PIMs was studied. Meanwhile, the physical–chemical properties of the modified PIMs were characterized via total reflectance Fourier transform infrared spectroscopy (ATR-FTIR), contact angle measurement, and electrical impedance spectroscopy (EIS). The relationships between transport flux and membranous physical–chemical properties of the modified PIMs would help the exploration of valuable clues to improve permeability efficiencies on the basis of membranous modification.

## 2. Experimental Section

### 2.1. Reagents and Chemicals

Poly(vinyl chloride) (PVC, MW~62,000) used as the PIM base polymer and 2-nitrophenyloctyl ether (NPOE, purity > 99%) used as plasticizer were purchased from Sigma-Aldrich. 2-hydroxy-5-nonylacetophenoneoxime (LIX^®^84I) and di(2-ethylhexyl)phosphoric acid (abbreviated as D_2_EHPA, purity > 95%) were provided by Cognis Co, Germany. Neodecanoic acid (Versatic acid 10, abbreviated as V10) was purchased from Shell Chemicals. Copper sulfate, ethanol, and sulfuric acid were purchased from Sinopharm, China. D_2_EHPA, V10, and ethanol were used as modifiers. Tetrahydrofuran (THF) was used as the solvent. All of the chemicals were used without further purification. The feed solution containing 1 mmol·L^−1^ Cu(II) was prepared by dissolving copper sulfate in ultrapure water. The pH value of the feeding solution was measured as approx. 4. Sulfuric acid solution with a concentration of 1 mol·L^−1^ was prepared as the receiving solution.

### 2.2. Preparation of PIMs

The PIM without the addition of a modifier (labeled as blank PIM) was prepared as reported previously [13,19]. For comparison, the modified PIMs in presence of modifiers (labeled as modified PIMs) were prepared in the following way: a 0.3 g mixture of PVC, NOPE, and LIX^®^84I with fixed mass fraction of 40%, 30% and 30% was dissolved in tetrahydrofuran (7 mL) under continuous magnetic stirring to obtain a homogeneous solution. Then, the desired amount of modifier was added into the homogeneous solutions. After stirring for 3 min, the mixed solution was then poured into a flat-bottom glass Petri dish with 7.5 cm in diameter, which was kept on a leveled surface, and covered with a filter paper to allow the slow evaporation of the solvent. The obtained PIMs were then carefully peeled off from the Petri dish and used for the Cu(II) transport experiments.

### 2.3. Transport Experiments

**Figure 1:**
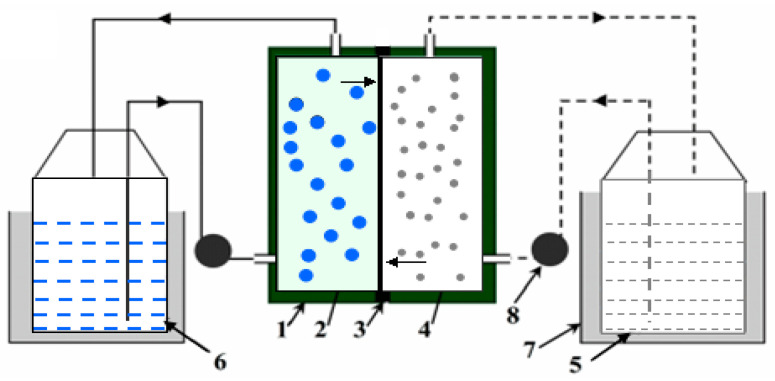
Schematic diagram of Cu(II) transport through PIMs. (1) Permeation cell; (2) Feeding solution; (3) PIMs; (4) Receiving solution; (5) Receiving solution; (6) Feeding solution; (7) Thermostatic water bath; (8) Peristaltic pump.

To evaluate the effect of the modifier components on the PIMs’ performance, membrane transport experiments were performed in a self-made two-compartment cell. The same volume of 100 mL was used for the feeding solution and the receiving solution, respectively, which is described in Figure 1. The temperature was kept at 30.0 ± 0.1 °C in a thermostat vessel. Both the feed solution and the striping solution were circulated by peristaltic pumps with a flow rate of 80 mL·min^−1^. Solution samples (1 mL) were taken periodically from both feed and receiving solutions for metal concentration analysis. Metal concentrations were analyzed by inductively coupled plasma atomic emission spectrometry (ICP-AES, Perkin Elmer 5300DV America).

The Cu(II) concentration within the membranous phase was calculated by mass balance. The transport behaviors of Cu(II) in the feeding phase, the receiving phase, and the membranous phase were calculated as follows:

The Cu(II) extraction in the feeding phase:

(1) $$Cu\left(II\right)\;extraction=\frac{{{\left[Cu(II)\right]}_{0}-[Cu(II)]}_{f,t}}{{\left[Cu(II)\right]}_{0}}\times 100\%.$$

The Cu(II) recovery in the receiving phase:

(2) $$Cu\left(II\right)\;recovery=\frac{{\left[Cu(II)\right]}_{s,t}}{{\left[Cu(II)\right]}_{0}}\times 100\%.$$

The Cu(II) retention in the membranous phase:

(3) $$Cu\left(II\right)\;retention=\frac{{{\left[Cu(II)\right]}_{0}-[Cu(II)]}_{f,t}-{\left[Cu(II)\right]}_{s,t}}{{\left[Cu(II)\right]}_{0}}\times 100\%.$$

The kinetics of the transport across the PIMs was described as a consecutive first-order reaction scheme, which is described in Reference [20].

(4) $$\mathrm{Cu}(\mathrm{II}{)}_{f}\overset{K_{1}}{\to }\mathrm{Cu}(\mathrm{II}{)}_{m}\overset{K_{2}}{\to }\mathrm{Cu}(\mathrm{II}{)}_{s},$$

where $K_{1}$ and $K_{2}$ are the rate constants for the feeding and the receiving reactions.

Considering the fact that Cu(II) transport from the feeding phase to the receiving phase would be virtually complete, in this kinetic behaviour, the variation rate of the metal reduced concentration in each phase can be described as

(5) $$\frac{{dR}_{f}}{dt}={-K}_{1}R_{f}=J_{f/m},$$

(6) $$\frac{{dR}_{m}}{dt}=K_{1}R_{f}{-K}_{2}R_{s}=J_{m},$$

(7) $$\frac{{dR}_{s}}{dt}=K_{2}R_{m}=J_{m/s},$$

where the dimensionless reduced concentrations of copper(II) in the feeding (*R_f_ = C_ft_/C_f_*_0_), membrane (*R_m_ = C_mt_/C_f_*_0_) and receiving (*Rs = C_st_/C_f_*_0_) phases are used (the sum of *R_f_* + *R_m_* + *R_s_* obviously being unity). $J_{f/m}$, $J_{m}$ and $J_{m/s}$ represent the instantaneous metal flux in each of the three phases. The integration of these differential equations lead to expressions of variations of $R_{f}$, $R_{m}$, $R_{s}$ with time. The time leading to the maximum value of $R_{m}$ can be obtained from $\frac{{dR}_{m}}{dt}=$ 0. Then, the maximum flux expressions can be expressed as

(8) $$[\frac{{dR}_{f}}{dt}{]}_{max}={{-K}_{1}\left(\frac{K_{1}}{K_{2}}\right)}^{\frac{{-K}_{1}}{K_{1}-K_{2}}}=J_{f}^{max},$$

(9) $$[\frac{{dR}_{s}}{dt}{]}_{max}={K_{2}(\frac{K_{1}}{K_{2}})}^{\frac{{-K}_{2}}{K_{1}-K_{2}}}=J_{s}^{max},$$

(10) $$-[\frac{{dR}_{f}}{dt}{]}_{max}=+[\frac{{dR}_{s}}{dt}{]}_{max}\underset{0}{\Rightarrow }-J_{f}^{max}=+J_{s}^{max}.$$

### 2.4. ATR-FTIR Spectra Studies

Attenuated total reflectance Fourier transform infrared (ATR-FTIR) spectra of the PIMs were recorded on a Nicolet 6700 spectrophotometer equipped with a Ge IRE crystal. The spectra were collected with a 45° take-off angle. The spectra of 4000–600 cm^−1^ with a resolution of 4 cm^−1^ were determined by accumulating 32 scans.

### 2.5. Contract Angles

The hydrophobic character of the PIM surfaces was determined by contact angle measurements which were performed by the tensile drop method using distilled water drops and Teclis T2010 instruments equipped with a video system. The average value of the contract angels for the three times was presented. Measurements were carried out on both pristine membrane (after evaporation) faces and covering a 2 cm × 2 cm area.

### 2.6. Electrical Impedance Spectroscopy Measurements

Electrical impedance spectroscopy (EIS) is a non-destructive alternating current technique, and it is commonly used for electrical characterization of systems. The impedance measurements of the PIMs were carried out at open-circuit potentials under potentiostatic control by using the CHI 760D electrochemical workstation (Shanghai Chenhua Instrument Co. Ltd., Shanghai, China). The test cell for the electrical impedance spectroscopy (EIS) consisted of two stainless steel meshes separated by two polyethylene rings that allowed the exposition of a 1 cm^2^ geometric area of the PIMs. The electrochemical impedance spectra were obtained from 0.01 Hz to 1 × 10^5^ Hz frequencies with a logarithmic collection of the data of 3 steps per decade and a maximum voltage of 14 mV. The impedance data were analyzed using the ZPlot and ZView software. Two physical parameters, including the electrical conductivity ($\sigma_{m}$) and dielectric constant ($\varepsilon_{m}$) which were directly related to the electrical response of a particular material could be determined by analyzing the impedance plots and using equivalent circuits as models [21,22]. The impedance of a system was a complex number, $Z=Z_{real}+{jZ}_{img}$, which could be separated into real and imaginary parts by algebra rules [22,23]. $Z_{real}$ and $Z_{img}$ were the electrical resistance and the capacitance calculated by the following expression:

(11) $$Z_{real}=(R/[1+{\left(\omega RC\right)}^{2}]).$$

(12) $$Z_{img}=-(\omega R^{2}C/[1+{\left(\omega RC\right)}^{2}]),$$

where ω is the angular frequency (ω=2πƒ). R and C denote the resistance and the capacitor obtained from an equivalent circuit.

## 3. Results and Discussion

### 3.1. Effect of Different Modifiers

**Figure 2:**
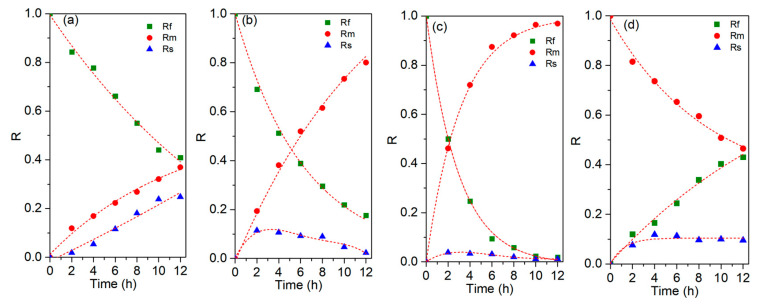
Time dependence of R_f_ in feed phase, R_m_ in membrane phase, and R_s_ in product phase in the facilitated counter transport of Cu(II) using the PIMs with and without modifiers. (**a**) D2EHPA, (**b**) ethanol, (**c**) V10, and (**d**) no modifier.

The effects of different modifiers in the PIMs on Cu(II) extraction, retention, and recovery efficiencies were studied. The added amounts of modifiers were labeled as *R_V_*_/*L*_, which was defined as the mass ratio of the modifier (abbreviated as *V*) to LIX^®^84I (abbreviated as *L*) in the PIMs. The rate constants of the extraction and stripping processes could be determined by non-linear curve fitting analysis of the transport experimental results (Table 1), and the model curve for the time dependence of $R_{f}$, $R_{m}$, $R_{s}$ (Figure 2) could be obtained. As shown in Figure 2, with the blank PIM, the extraction efficiency of Cu(II) is 53.5%, and the recovery efficiency of Cu(II) is 45.9%, which means that around 7.6% of Cu(II) is kept in the membranous phase. The chelation reaction of Cu(II) and the carrier LIX^®^84I(HA) offers driving force for Cu(II) to be extracted into the membrane phase, according to the following Equation [24]:

(13) $${{Cu}^{2+}}_{\left(aq\right)}+2\bar{{\left(HA\right)}_{\left(PIM\right)}}↔\bar{{\left(Cu\cdot 2A\right)}_{\left(PIM\right)}}+2{H^{+}}_{\left(aq\right)}.$$

**Figure 3:**
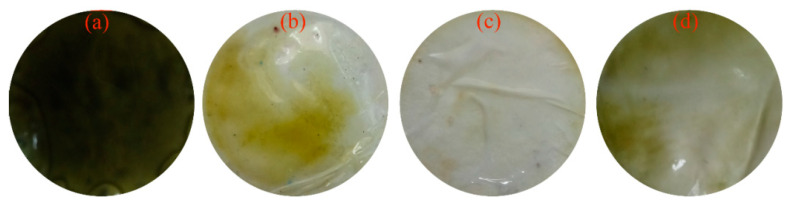
Digital images of the membranous surfaces of the PIMs with and without modifiers. (**a**) D2EHPA, (**b**) ethanol, (**c**) V10, and (**d**) no modifier.

Obviously, the migration of the Cu(II) complex within the membrane might be decreased because of the difficulty of solid diffusion within the membranous phase. In addition, the phase interface between the membrane and the solution hinders the reaction of hydrophilic Cu(II) and the hydrophobic LIX^®^84I because of the differences in their hydrophilicity. This caused that membranous resistance might be the reason for the inefficiency transport of Cu(II). To verify this hypothesis, some modifiers were added into the LIX^®^84I-based PIMs, and their transport behaviors of Cu(II) were compared. While cast with the di(2-ethylhexyl)phosphoric acid (D_2_EHPA) as a modifier, the extraction of Cu(II) is slightly improved, but the back extraction of Cu(II) is depressed (shown in Figure 2a). At 12 h, approx. 62.8% of Cu(II) is extracted from the feed solution, while the receiving efficiency of Cu(II) is approx. 37.9%. The Cu(II) retention efficiency in the D_2_EHPA-modified PIM reaches ~24.9%. The successive accumulation phenomenon of Cu(II) in the D_2_EHPA-modified PIM indicates that the addition of D_2_EHPA deteriorates the transport of Cu(II). This phenomenon can also be verified from the observation of a dark green membrane after Cu(II) transport (Figure 3a), indicating that a amount of copper complexes are accumulated in the PIM matrix.

As shown in Table 1, the rate constants for Cu(II) extraction and stripping processes by using the unmodified PIMs are 0.69 h^−1^ and 0.92 h^−1^. With the addition of D2EHPA, the metal flux of Cu(II) is obviously enhanced. The rate constant for Cu(II) extraction process is increased to 1.31 h^−1^. However, the rate constant for the Cu(II) stripping process is reduced to 0.55 h^−1^. When ethanol or V10 is added into the PIM as the modifier, the metal flux of Cu(II) is obviously enhanced. After using the ethanol as the modifier, the rate constants for Cu(II) extraction and stripping processes are increased to 0.90 h^−1^ and 1.17 h^−1^, respectively. In addition, the rate constants for the Cu(II) extraction and stripping process with the V10-modified PIMs are 1.00 h^−1^ and 1.01 h^−1^, respectively. Compared with the unmodified PIMs, the maximum flux of Cu(II) with the modified PIMs using ethanol or V10 is increased from 0.29 h^−1^ to 0.37 h^−1^. The results indicate that the addition of ethanol or V10 can improve the transport flux of Cu(II). The results are in accordance with the previously reported findings [13,23]. When V10 is added into the PIMs, the extraction, recovery, and retention efficiencies of Cu(II) are 98.2%, 97.0%, and 1.2%, respectively. It is interesting to observe that the addition of V10 can also evidently reduce the Cu(II) retention and redound Cu(II) permeability. From Figure 3c, it can be observed that there are almost no copper complexes left within the PIM. It can be seen that the Cu(II) transport is greatly improved by the modified PIMs with the addition of Versatic10. Based on the evidenced transport behaviors of the PIMs cast with different modifiers, PIMs in presence of Versatic acid 10 are selected to further explore the modification effects on the transport flux of Cu(II) and their physical–chemical characteristics.

### 3.2. Effects of V10 Content on the Transport Behaviors

**Figure 4:**
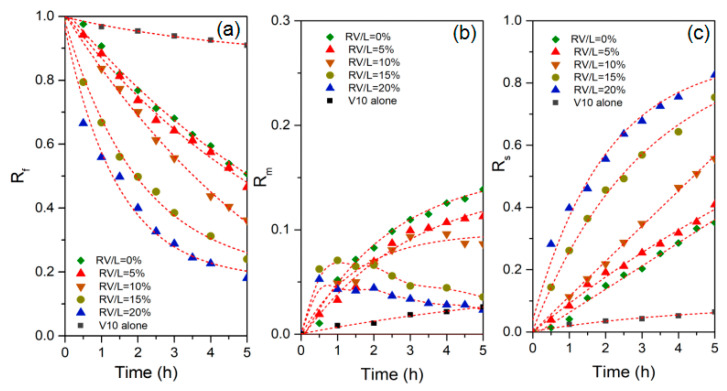
Effect of V10 content on (**a**) R_f_ in feed phase, (**b**) R_m_ in membrane phase, and (**c**) R_s_ in product phase of Cu(II) through the V10-modified PIMs.

In the literature, some researchers used Versatic 10 as one of the binary carriers within a PIM to improve the receiving efficiency of Sc^3+^ [24]. They considered that the presence of Versatic 10 would reduce the affinity of PC-88A to Sc^3+^ so as to enhance the membranous transmission. In order to further explore the effects of Versatic 10 on membranous transport flux, different V10 contents within the LIX^®^-based PIMs on the extraction and recovery efficiencies were examined.The results are shown in Figure 4. It is observed that with the increase in the V10 content, both the extraction and recovery efficiencies of Cu(II) increase, while the membranous retention decreases. When the additional content of V10 (*R_V_*_/*L*_) increases from 0% to 20%, the Cu(II) extraction efficiencies increase from 46.1% to 84.9%, and the Cu(II) extraction efficiencies increase from 35.1% to 84.9% to 82.6% at 5 h (Figure 4). Accordingly, the maximum accumulation amount of Cu(II) almost decreases from ~10% to ~5%. As mentioned in Section 3.1, the Cu(II) transport process is generally effected by three stages, including extraction reaction between Cu(II) and carriers at the feed solution/interface of PIMs, diffusion of Cu(II) complex across PIMs and dissociation of the complex by hydrogen ions at the interface of the PIM/receiving solution. To understand the reason for the enhanced transport phenomena, the modified membranous structural properties are further explored.

### 3.3. ATR-FTIR Analysis

**Figure 5:**
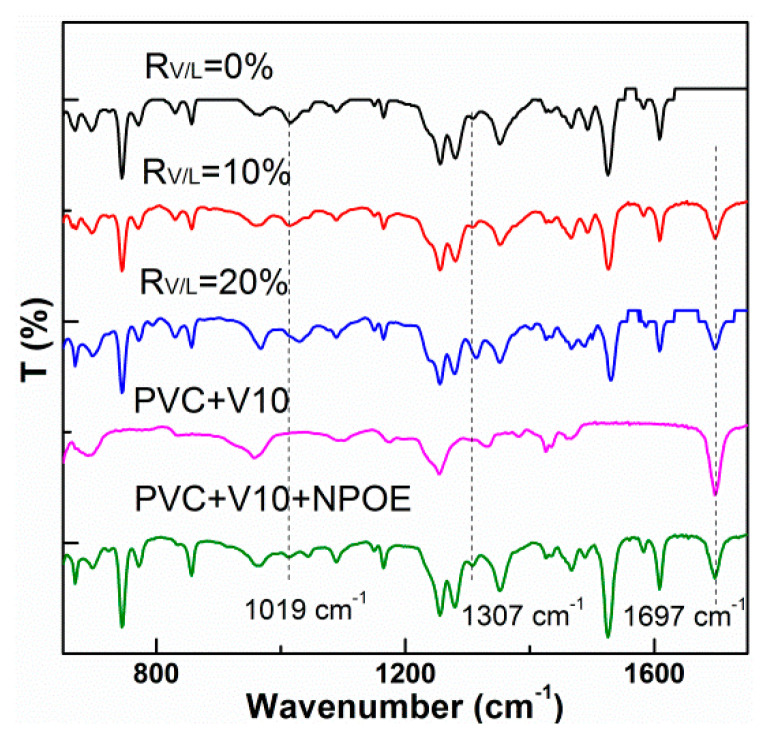
ATR−FTIR spectra of the modified PIMs with V10.

In order to elucidate the possible interactions between the base polymer, plasticizer, carrier and modifiers, ATR-FTIR spectra of PIMs were investigated. The main spectral feature of the membrane composed of PVC and V10 in Figure 5 is the characteristic peak located around 1697 cm^−1^, which is attributed to the stretching vibration of the C=O group in the V10 molecules. In addition, the characteristic peak located at 1390–1330 cm^−1^ is assigned to the stretching vibration of the O-H group in the V10 molecules [25]. The strong peaks located around 1525 cm^−1^ and 1164–960 cm^−1^ are attributed to the vibrations of the C–O–CH_2_ groups in NPOE. It is worth noting that that after adding V10 as the modifier, the peaks at 1019 and 1307 cm^−1^, which are assigned to the C-NO_2_ asymmetric vibrations of the NPOE molecules, present a slight red shift. In addition, the peak intensities were enhanced when comparing the PIM with *R_V_*_/*L*_ of 0% and the PIM with *R_V_*_/*L*_ of 20% in Figure 5. This change implies the bonding between the plasticizer NPOE and modifier V10 [26]. Especially the plasticization of the NPOE molecules depends on their interpenetrating in the PVC skeleton, which decreases the intermolecular force of PVC chains and induces the enhancement in the flexibility of PIM. Therefore, based on the IR analysis, the addition of V10 potentially improve the plasticization of the NPOE due to the interactions between the membranous components.

### 3.4. Contact Angle Analysis

**Figure 6:**
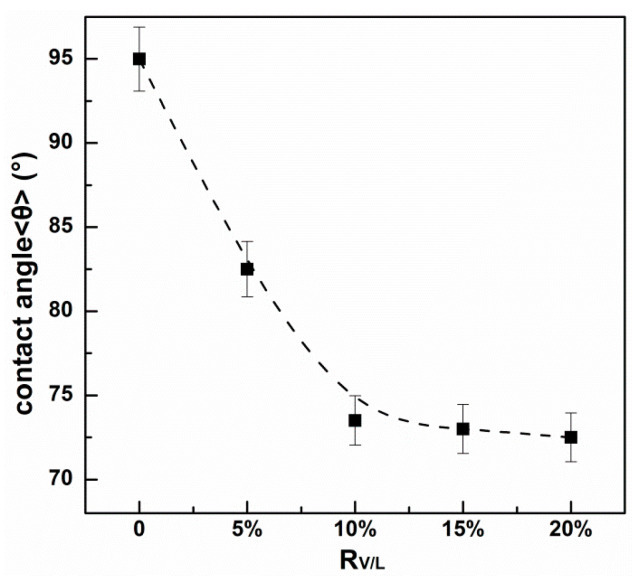
Variation of contact angles of the modified PIMs with the different V10 contents.

A parameter related to physico-chemical properties in the membrane materials is the contact angle, which provides information on their hydrophobic/hydrophilic character. Figure 6 shows a comparison of the average values obtained for the modified PIMs at the different V10 contents. These results correspond to the average value of the three measurements of both surfaces for each membrane (deviation of lower than 5%). It can be seen that the contact angle of the unmodified PIM is around 95°, indicating a hydrophobic surface of the PIM. In particular, the hydrophobicity of the modified PIM greatly reduces. The contact angle of the V10-modified PIM decreases to about 73° when the *R_V_*_/*L*_ is above 10%, indicating that the surface of the modified PIM changes from hydrophobic to hydrophilic. The transition of hydrophobic/hydrophilic characters might be ascribed to the introduction of the polar hydrophilic functional groups (e.g., -COOH) into the membranous phase. Given that the hydrophilic character of the modified PIMs correlates with the increase trend of membrane permeability and reduction in metal accumulation, it was assumed that the hydrophilicity might alleviate the initial resistance absorption of Cu(II) species on the membrane surface and allow a solvating environment appropriate for ion diffusion [27]. In the previous studies, R. Vera also suggested that the higher hydrophilicity of the PIMs benefited the ion diffusive transport by varying the corresponding counter anion [28].

### 3.5. EIS Analysis

**Figure 7:**
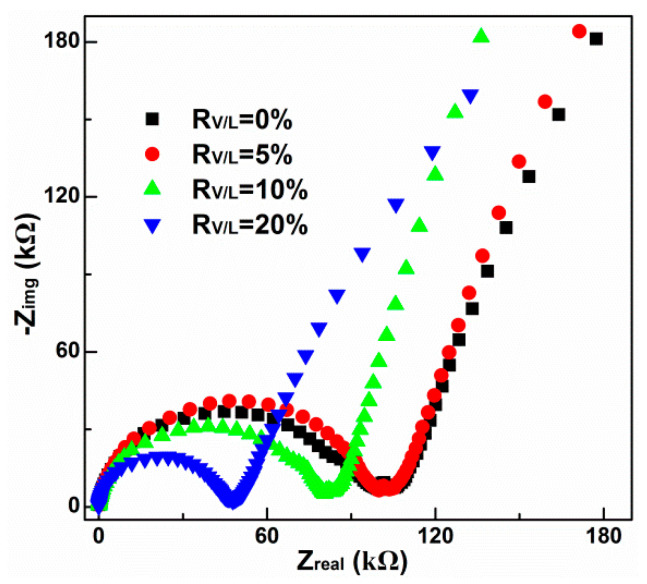
Nyquist plots (−Z_img_ versus Z_real_) of the modified PIMs with different V10 contents.

In order to establish a correlation of electrical response and the membranous modification, the modified PIMs were further characterized by electrical impedance spectroscopy (EIS). Figure 7 displays a comparison of the Nyquist plots obtained for the PIMs with different V10 contents. Only one relaxation process (one semicircle in the Nyquist plot) is observed for the four tested PIMs. The semicircles obtained with the four PIMs indicate a direct correlation between the electrical resistance and the V10 contents. As seen in Figure 7, the resistance reduces with the increase in the V10 contents. The unmodified PIM shows the highest overall resistance, while the PIM cast with a 20% V10 reduces by half (compare the relative diameters of the semicircles shown in Figure 7). In addition, it is observed that for all the systems, the order of the tested membrane resistance is in excellent agreement with the extraction order of Cu(II) obtained from transport experiments (Section 3.2). These findings actually help to understand the facilitation of membranous modification as the lower the membranous resistance, the higher the transport flux of Cu(II).

Another important parameters which are able to shed information on bulk membrane modifications associated to V10 contents are the electrical conductivity and the dielectric constant [29]. For this reason, membrane conductivity $\left(\sigma_{m}\right)$ and dielectric constant $\left(\varepsilon_{m}\right)$ are determined based on Equations (14) and (15) by taking into account the expressions for homogeneous conductors and plane plate capacitors, respectively [20,30]:

(14) $$\sigma_{m}=\frac{\Delta \chi }{R_{m}S},$$

(15) $$\varepsilon_{m}=\frac{C_{m}\Delta \chi }{S\varepsilon_{0}},$$

where Δχ, S, and $\varepsilon_{0}$, represent the membrane thickness, membrane area, and the vacuum permittivity, respectively. *R_m_* and $C_{m}$ represent the membranous electrical resistance and equivalent capacitance, respectively.

**Figure 8:**
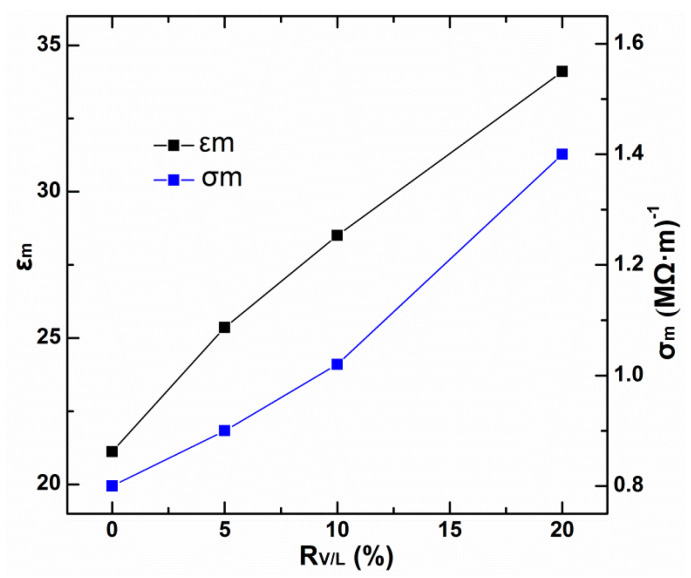
Variation of membrane dielectric constants ($\varepsilon_{m}$) and membrane conductivities ($\sigma_{m}$) for the modified PIMs with different V10 contents.

Variations of $\sigma_{m}$ and $\varepsilon_{m}$ with the V10 content are represented in Figure 8. It can be seen that the increase in V 10 content clearly increases both the membrane dielectric constant and the conductivity independent of the membrane supporting material.

As can be observed, the addition of V10 into the PIMs increases the membrane conductivity, suggesting that a significant contribution is made by the V10 modifier in such a formulation (Figure 8). The results indicate that the dielectric constants of the resulted PIMs vary with the increase in the V10 content. When the *R_V_*_/*L*_ reaches 20%, the dielectric constant of the modified PIMs increases to 34.5, which is much higher than that of 2-nitrophenyl octylether (around 24) [30] and polymers alone (ranging between 2 and 8) [31]. These results provide evidence for the modification of V10 as on the polar characters of PIMs as well as their conductive behaviors. In fact, the dielectric constant ($\varepsilon_{m}$) parameter influences the balance between the efficiency of association and dissociation steps for the uptake of the metal ion by the carrier and its release from the complex at the receiving interface [19,29,32]. As suggested by Fontas et al., the carrier was solvated by the plasticizer, which acted as a solvating medium, favoring the occurrence of liquid micro-domains [33]. The disruption in the transport pathway is a function of both the physical dispersion of the components and of their mobility. The enhanced diffusion behavior of Cu(II) across the modified PIMs might be explained by the assumption that the more hydrophilic surface allows better accessibility of metal ions to the membrane, thereby the diffusion resistance is decreased and the carriers are be sufficiently engaged in the transporting process.

## 4. Conclusions

This study reported a possible modification method for facilitating the transport flux of Cu(II) through the LIX^®^-based PIMs. The transport data indicated that the modified LIX^®^-based PIMs showed considerably higher permeability, achieving faster transport efficiency and less membranous retention. In addition, the physical–chemical characteristics of the modified LIX^®^-based PIMs were revealed via characterization using ATR-FTIR spectra, contact angles and EIS measurement. It was worth mentioning that the hydrophilic/hydrophobic character of the LIX^®^-based PIMs could be tuned by the use of modifiers. In addition, higher conductivity and dielectric constants were found for the modified PIMs. Given this, we ascribed the enhanced transport flux of the modified PIMs to the change in PIM membranous physicochemical properties. As such, a highly possible way for the development of efficient polymer inclusion membranes could be provided and therefore prompt their potential applications.

## Figures and Tables

**Table 1:** Rate constants for extraction and stripping processes, maximum flux ($J_{0}^{max}$), extraction efficiency, and recovery efficiency (SE) for the coupled transport of Cu(II) using different modified PIMs.

Modifier	$K_{1}$ $\left(h^{-1}\right)$	$K_{2}$ $\left(h^{-1}\right)$	$J_{max}$ $\left(h^{-1}\right)$	Extraction (%)	Recovery (%)
D_2_EHPA	1.31	0.55	0.29	62.75	37.96
Ethanol	0.90	1.17	0.37	84.08	81.78
V10	1.00	1.01	0.37	98.21	97.02
None	0.69	0.92	0.29	53.51	45.99

## Data Availability

The data presented in this study are available on request from the corresponding authors.
